# Functional Testing of Vestibulo-Spinal Contributions to Balance Control: Insights From Tracking Improvement Following Acute Bilateral Peripheral Vestibular Loss

**DOI:** 10.3389/fneur.2019.00550

**Published:** 2019-05-28

**Authors:** John H. J. Allum, Heiko Mario Rust, Flurin Honegger

**Affiliations:** ^1^Division of Audiology and Neurootology, Department of Otorhinolaryngology (ORL), University Hospital Basel, Basel, Switzerland; ^2^Department of Neurology, University of Basel Hospital, Basel, Switzerland; ^3^Division of Brain Sciences, Academic Department of Neuro-Otology, Charing Cross Hospital, Imperial College, London, United Kingdom

**Keywords:** bilateral vestibular loss, posturography, vestibulo-spinal reflexes, vestibular evoked, vemps, vestibulo-ocular reflexes, video head impulse test

## Abstract

**Background:** A battery of stance and gait tasks can be used to quantify functional deficits and track improvement in balance control following peripheral vestibular loss. An improvement could be due to at least 3 processes: partial peripheral recovery of sensory responses eliciting canal or otolith driven vestibular reflexes; central compensation of vestibular reflex gains, including substitution of intact otolith responses for pathological canal responses; or sensory substitution of visual and proprioceptive inputs for vestibular contributions to balance control.

**Results:** We describe the presumed action of all 3 processes observed for a case of sudden incapacitating acute bilateral peripheral loss probably due to vestibular neuritis. Otolith responses were largely unaffected. However, pathological decreases in all canal-driven vestibular ocular reflex (VOR) gains were observed. After 3 months of vestibular rehabilitation, balance control was normal but VOR gains remained low.

**Conclusions:** This case illustrates the difficulty in predicting balance control improvements from tests of the 10 vestibular end organs and emphasizes the need to test balance control function directly in order to determine if balance control has improved and is normal again despite remaining vestibular sensory deficits. This case also illustrates that the presence of residual otolithic function may be crucial for balance control improvement in cases of bilateral vestibular hypofunction.

## Introduction

It is an open question whether any improvement in balance control following an acute bilateral peripheral vestibular loss (BVL) uses the same neural processes to improve function as when the acute peripheral vestibular loss is unilateral (UVL). An acute UVL resulting from presumed vestibular neuritis (VN) causes a characteristic deficit in the vestibular ocular reflex (VOR) function easily observed with a head impulse test of the deficit side VOR by the presence of catch-up saccades and a gain < 0.74 (1). Acutely, no or little change in VOR function is observed with a video head impulse test (vHIT) for head rotations to the normal side (gain equal to 1). A gain reduction to 0.5 on average occurs for rotations to the deficit side (2). Insights into the neural processes underlying central compensation for the UVL deficit side gain can be obtained by examining a group of acute UVL patients who have a large, almost complete, lateral canal loss as determined with caloric testing [that is, a 90% and greater canal paresis (CP)], and also no CP recovery over the following 3 months. Despite no CP recovery, that is, no recovery of the peripheral sensory function, the vHIT deficit side gains improve on average in this group from 0.45 in the acute state to 0.64 over the following 3 months. It is assumed that this improvement is due to central compensation, specifically, that the crossed intact side input contributing to the VOR for head rotations to the deficit side is enhanced (3–5). The question thus arises when there is no recovery of peripheral vestibular function *bilaterally*, as indicated by absent caloric responses, and abnormally low canal-elicited vHIT responses at acute onset, that is a gain below 0.6 bilaterally, [see Strupp et al. for a consensus definition of BVL (6)] whether there can be any improvement in VOR gains and balance control. In this situation, there is no normal side response to aid central compensation.

Long-term (< 3 months) differences in peripheral vestibular recovery, which may be observed as differences in vHIT gains following an acute UVL, do not lead to long-term differences in balance control during stance and gait tests (5, 7). For example, for the stance test most sensitive to an acute UVL, standing eyes closed on foam (8), there is no significant difference in trunk sway for those with and without peripheral vestibular recovery after 3 months (5). Sway for both groups is, on average, normal. This is not the case for patients with chronic bilateral vestibular loss (BVL) (9). These patients still have sway velocities and angles greater than normal (9). For the gait test most sensitive to an acute UVL, walking eyes closed (8, 10), differences do emerge, trunk roll angle and pitch velocity is larger and greater than normal for the non-recovery group (5). There are several possible reasons for these differences in functional deficits which could have application to cases with an acute BVL. Firstly, there is a difference in recovery times for stance and gait tasks, with stance tasks recovering more rapidly after an acute UVL (11), possibly because VOR recovery is more rapid for the slow vs. the high head accelerations which occur with stance and gait, respectively (11, 12). Secondly, central compensation increasing the use of visual contributions to balance control could restrict the tests with differences to normal values to those performed with eyes closed. Nonetheless, even with eyes closed, the effect of central compensation increasing the use of somatosensory inputs could help improve balance stability.

There are other more fundamental problems involved with predicting balance capabilities from VOR responses in canal planes. Given the generally weak correlations between vHIT results and balance measures (7), except for roll during gait (13), the question arises how to determine the relationship between weakened canal VOR responses and vestibulo-spinal contributions to roll and pitch balance control during stance and gait. As spared function of the otoliths could be used by the CNS to generate angular velocity based sensory inputs no longer provided by canal reflexes (14, 15), it would be important to include tests of otolith function in the determination of these relationships.

Otolith function is usually measured using vestibular evoked myogenic potentials (VEMPs) elicited by sound-induced movements of the otoliths from the sternocleidomastoid (SCM) and inferior oblique (Inf Obl) muscles (16, 17). It is difficult to translate VEMP responses, if pathological, into deficits in balance control for three reasons: firstly, because it is difficult to elicit VEMPs from muscles such as soleus, tibialis anterior, and paraspinals (18) that are involved in balance control; secondly, because the amplitude polarity of vestibulo-spinal contributions of muscle responses elicited by perturbations to stance of these muscles differ across the body (19, 20) as does the amplitude polarity of VEMP responses to tone bursts in different muscles (16): thirdly, because the VEMP waveforms are often regarded as present or absent, except in the case of vertical canal dehiscence (21), the strength of the response does not necessarily translate into a strength or degree of function or dysfunction. Thus, although SCM and Inf Obl VEMPs provide an insight into the status of otolith sensory inputs to vestibular spinal control, these are unlikely to replace functional tests of the influence of vestibule-spinal signals on balance control.

In the current report, we emphasize the importance of recording VEMPs as these may provide insights into otolith-based balance improvements, in addition to insights into canal-based improvements revealed by the amount of recovery in vHIT examinations. While testing for the status of sensory contributions to balance control is important, we consider it crucial to ascertain the status of balance control with appropriate stance and gait tests. In this reported case of a sudden acute BVL we were impressed with the remarkable recovery in balance control despite the weak improvement in all canal VOR responses. In stark contrast, sacculus c-VEMP responses were normal, and utriculus o-VEMP responses were only weaker than normal on the right side. This report differs from a previous report of an acute BVL patient with only modest improvement in patient symptoms (22) in 3 aspects. Firstly, in our patient the loss involved all canal VOR responses and not just those served by the superior vestibular nerve (lateral and anterior canals). Secondly, our patient had remaining otolith function for all 4 otoliths. Thirdly, by tracking balance control we were able to document its remarkable improvement, matching improvement in patient symptoms.

## Methods

The current case concerns a male, 49 years old high rise crane driver who was suddenly incapacitated with vertigo (both tilting and turning) and nausea in his crane cockpit and had to be rescued with another crane. Initially, he could not walk without assistance. The patient and his general practitioner reported no prior deficit of balance control and no medical history consistent with a previous vestibular sensory deficit. Previous periodic testing of hearing by the Swiss Accident Insurance also revealed no deficits. Such testing is mandatory in Switzerland for high-rise crane drivers. The lateral canal vHIT gains measured on entry to our hospital's emergency ward were 0.27 right and 0.43 left. Hearing was normal. A neurological examination on the same day revealed no other abnormalities. Specifically, a 3 Tesla MRI on the day of admission and 7 months later showed no signs of an ischemic attack. Based on the test results consistent with vestibular neuritis, the patient was treated intravenously with methylprednisolone (125 mg Solumedrol™ per day) and then on discharge 6 days after entry with the oral medication. The patient received sessions of balance-oriented physical therapy daily while an in-patient and twice weekly with muscle conditioning for 9 months on discharge. Tests of optokinetic nystagmus, smooth pursuit tracking, and saccades performed 8 days after initial onset of the symptoms were normal except for a bias in the optokinetic nystagmus tests approximately equal to the level of spontaneous nystagmus (4 deg/s).

Scientific use of the data collected for this study was approved (approval 2014–16) by the local ethics committee responsible for the University Hospital Basel [Ethics Committee Northwest and Central Switzerland (EKNZ)]. Written informed consent was obtained from the acute BVL patient for the publication of his data from routine clinical examinations to be presented in this report.

### VOR Measures

**Figure 1:**
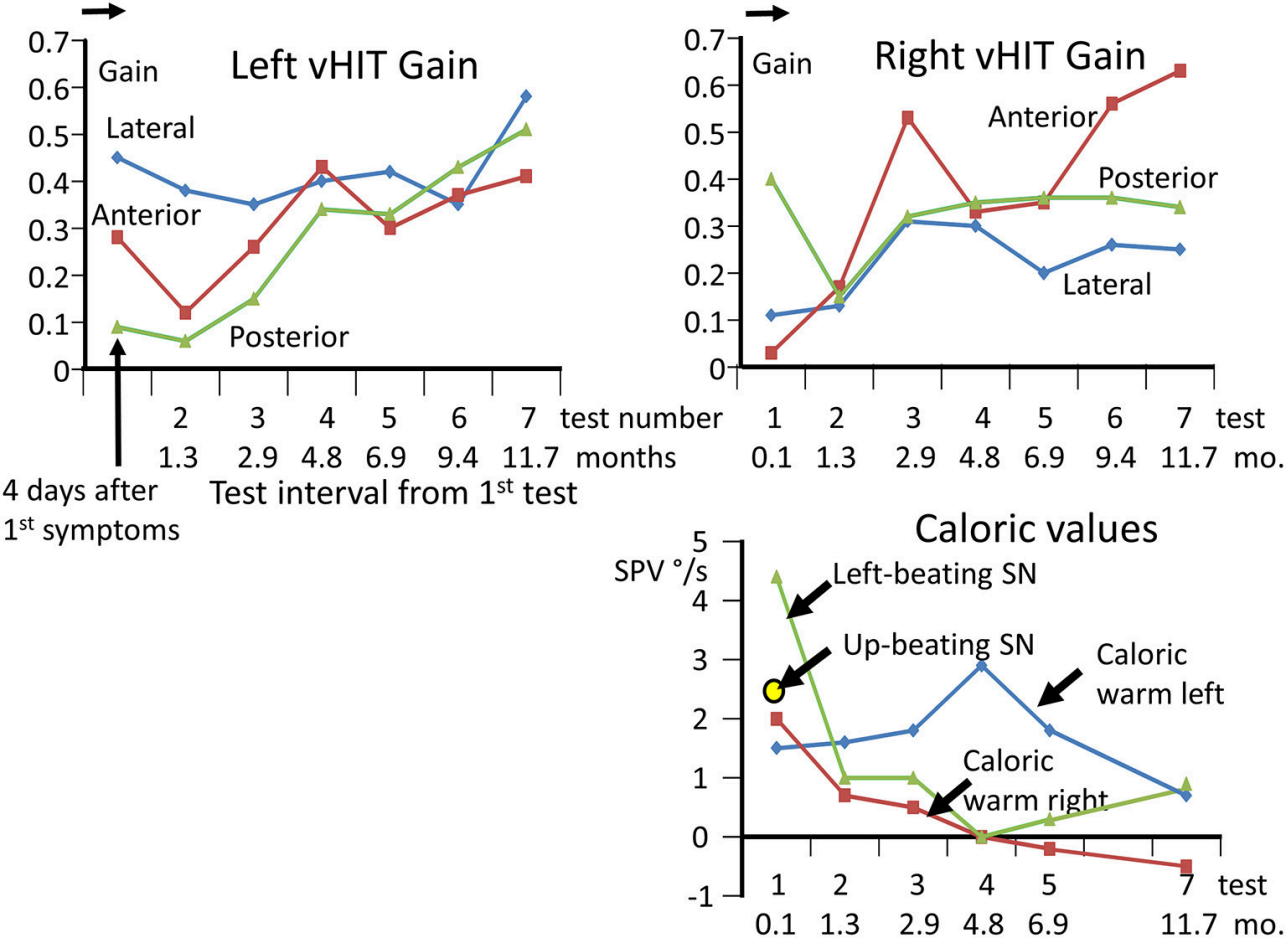
Upper panels: changes in vHIT gains in each canal direction, from 4 days after onset of acute symptoms until 11.7 months later. A total of 7 tests were performed. Note the gradual improvement in the vHIT gains except for the left lateral and right posterior gain. All gains are lower than normal as marked by the arrow on the gain ordinate, indicating the lower 95% limit of healthy controls (23). Lower right panel: caloric measurement values over time and levels of spontaneous nystagmus. The values for the nystagmus slow phase velocity (SPV) elicited during the culmination phase 60–80 s from the start of a 60 s irrigation of 44°C are listed after subtraction of the SPV of the left-beating spontaneous nystagmus recorded over 20 s prior to the irrigation. Because of the small values, the canal paresis was not calculated. For the examination at 4 days only, an up-beating vertical spontaneous nystagmus was also observed.

Canal paresis measures could not be determined using a bithermal (44 and 30°C) caloric test due to the very low responses (see Figure 1). Instead, only the average eye slow phase velocity (SPV) over the culmination phases of nystagmus was computed for the left and right ear irrigations at 44°C (see Figure 1)^1^. To measure VOR function in response to high angular accelerations (above 2,000°/s^2^) a video head impulse test (vHIT) system was used (ICS system from GN Otometrics) according to the protocol described by MacDougall et al. (24) with head angular velocities reaching 80–250°/s by 100 ms. At least 20 head lateral rotations to each side and in the planes of each vertical semi-circular canals were performed. During the head movements, the patient was seated and fixed gaze on a small target 3 m away. Sections of the data with covert saccades and artifacts were removed from the recordings prior to gain calculations by the vHIT manufacturer's software. Gains were calculated based on the quotient of the areas under the eye and head velocity impulse responses. The interval used started 100 ms prior to peak head velocity and ended when head velocity first crossed zero after the peak. In the emergency ward, the patient's first vHITs were measured with an ESC system (Interacoustics). Gain values are computed differently with this system compared to the ICS system we used for all subsequent vHITs. For this reason, the gain ESC gain values were converted to equivalent ICS gains using the technique described in Cleworth et al. (25).

### Balance Control Measures

Balance control was assessed by measuring trunk sway during a sequence of 14 stance and gait tasks. All stance and gait tasks were performed in the same order and executed without shoes. The tasks used were chosen based on our previous studies comparing balance for the 14 stance and gait balance tasks between different patient groups and healthy controls (10, 26). The same protocol is also used for routine clinical balance control examinations in our clinic. Trunk sway during the tasks was measured with a SwayStar™ device (Balance International Innovations GmbH, Switzerland) which uses two gyroscopes to measure pitch (anterior-posterior) and roll (lateral) angular velocities of the lower trunk at a sample rate of 100 Hz. Angles were determined on-line by trapezoid integration of the velocity signals. The device is worn in the middle of the lower back of the patient to be tested (at the level of lumbar vertebrae L3–L5) near the body's center of mass (10). The SwayStar™ device has been validated by a number of clinical studies, specifically on patient groups affected by vestibular loss (8, 10, 11), and allows comparison with a normal reference data set (26).

Four 2-legged balance tests were performed with the feet spaced shoulder width apart. Two were performed with eyes open, on a normal surface and on a foam surface (height 10 cm, density 25 kg/m^3^), and 2 with eyes closed (abbreviated s2eo/s2ec/s2eof/s2ecf). Three 1-legged stance tasks were performed eyes open, two on a normal surface (eyes open and eyes closed) and one, eyes open, on the foam surface (s1eo/s1ec/s1eof). For the 1-legged tasks, the patient was asked to stand on their preferred leg. The stance tasks were performed on foam to reduce the contribution of lower-leg proprioceptive inputs to balance control. Stance tasks were performed for 20 s or until the patient lost balance. The patient performed 2 tandem gait tasks: walking 8 tandem steps on a normal and foam surface (w8tan/w8tanf), and 3 walking tasks: walking 3 m while pitching the head up and down with eyes open (w3mhp); while rotating the head left and right with eyes open (w3mhr) and walking 3 m, eyes closed (w3mec). Tasks were performed with eyes closed to eliminate visual inputs to balance control. For gait tasks, the patient was asked to walk at their comfortable pace. Finally, the patient was asked to walk up and down a set of 3 stairs (constructed similar to a podium), and walk over 4 low (24 cm) barriers spaced 1 m apart. For gait tasks, the task duration was the time it took to complete the task or until the patient lost balance and needed to be assisted by a spotter. To standardize the start of each gait task, the patient was asked to stand comfortably with feet hip-width apart.

### Vestibular-Evoked Myogenic Potentials (VEMPs)

VEMPs were elicited from the sternocleidomastoid (SCM) and inferior oblique (Inf Obl) muscles using 5 ms duration air-conducted 500 Hz tone bursts (rise and fall times 2 ms) and delivered at a rate of 5.1 Hz. Normally the test amplitude was 85 dB normal hearing level. This level was increased or decreased depending on whether a response was observed at 85 dB. All muscles were tonically active during the experiments to ensure a VEMP response was elicited. The SCM was activated by having the patient voluntarily maintain a yaw head rotation 60° to the left for right ear stimulation, and vice-versa for the left ear. The Inf Obl was activated by having the patient look upwards. Averages to 500 stimuli were computed after high and low pass filtering at 10 and 1,500 Hz, respectively. From these averages peak-to-peak p13 to n23 amplitudes for c-VEMPs and n1 to p1 for o-VEMPs were compared with the lower 95% amplitude of normal responses (27). For further details, see the legend to **Figure 4**.

### Data Processing and Statistical Analysis

**Figure 2:**
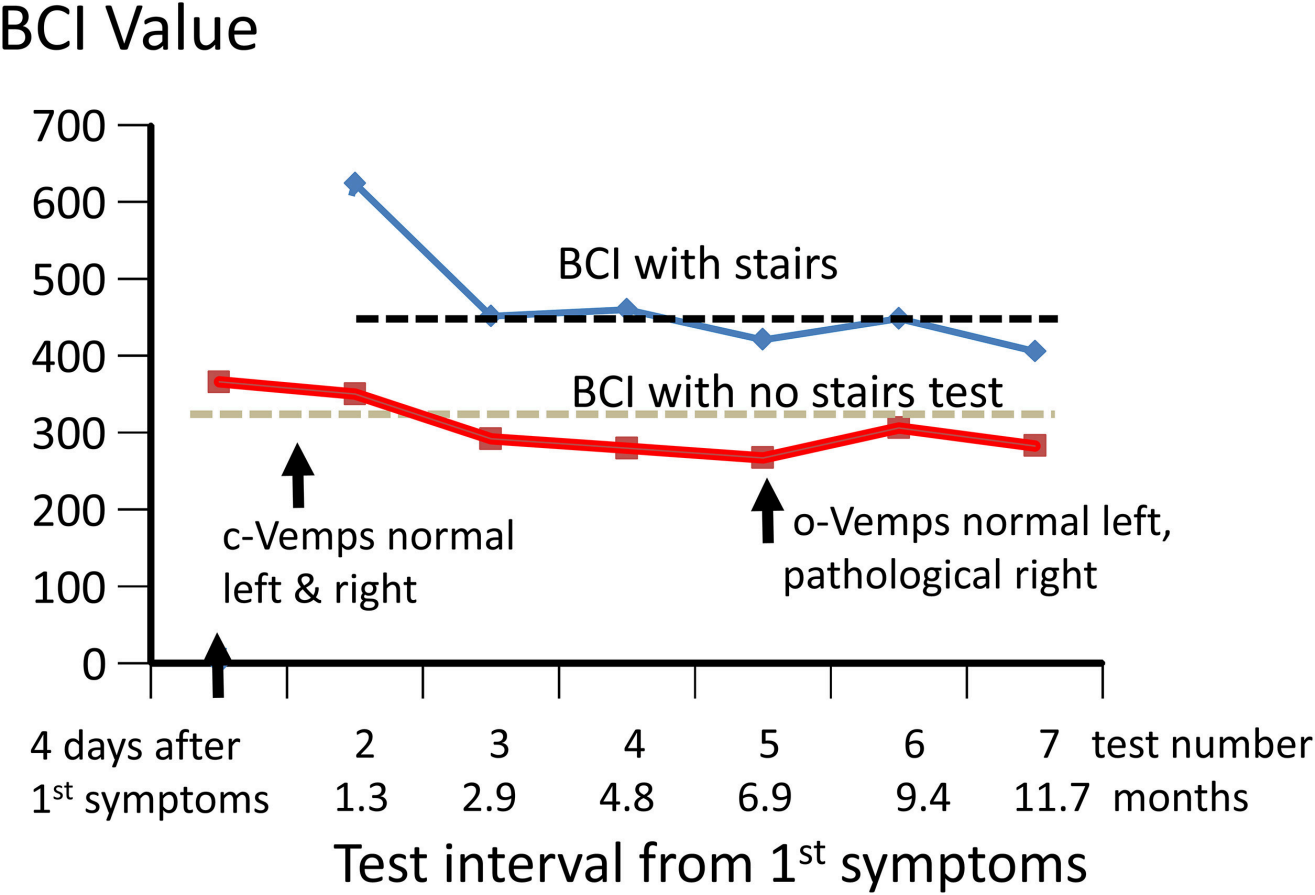
Changes in Balance Control Index (BCI) over 12 months from onset of acute symptoms. The values of the BCI with and without the stairs test included are shown. The stairs test could not be performed for the first test due to the instability of the patient. The upper 95% limit of BCI values (lower values more normal) for the 54 healthy subjects whose ages were within ± 5 years of that of the patient (49 years) are shown by the dashed black and gray lines. The times the c- and o-VEMPs were measured are also indicated.

**Figure 3:**
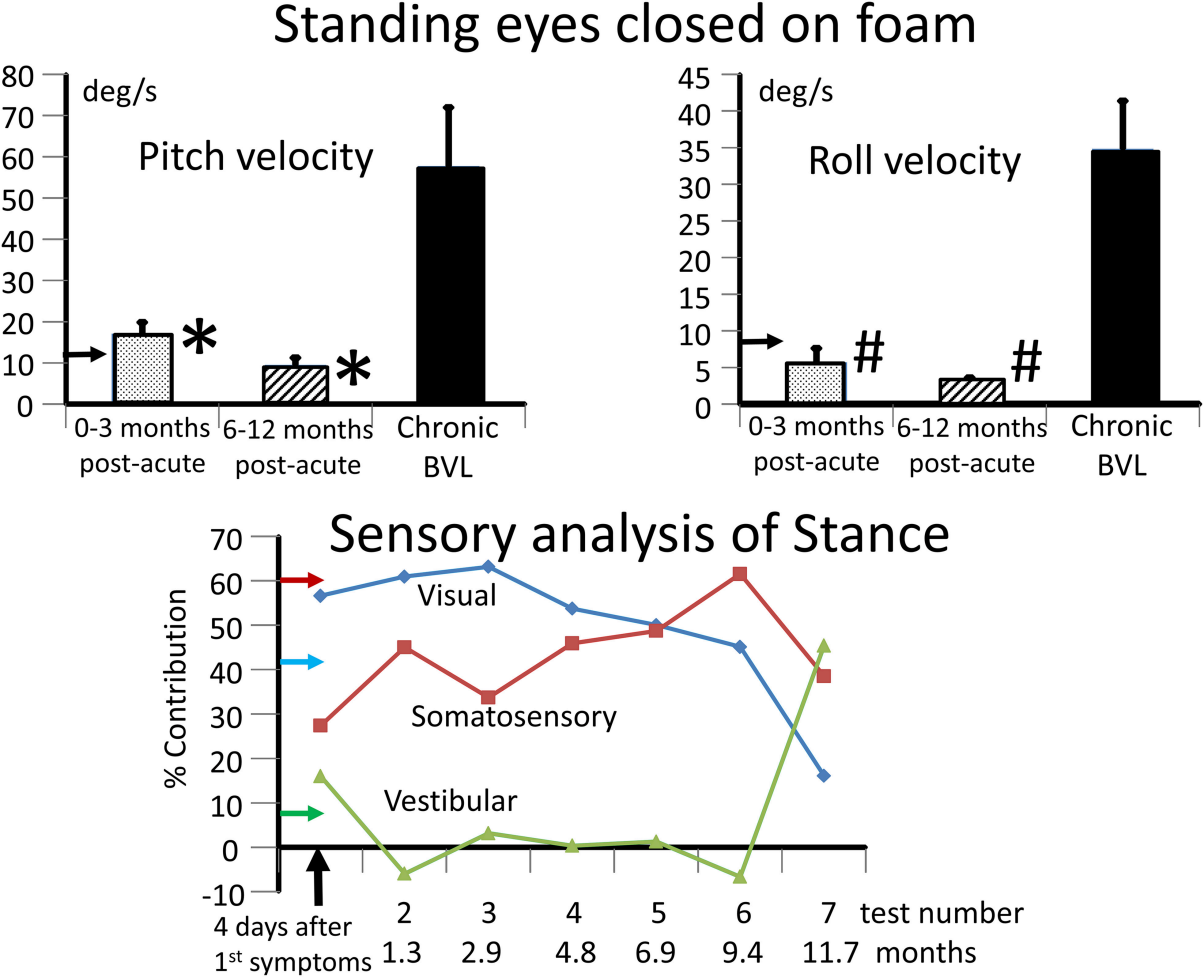
Upper panels: mean values and standard error of mean (sem) of BVL subjects for the range of trunk pitch and roll velocity while standing eyes closed on a foam surface. The leftmost column in each panel depicts the mean of 3 tests in the 3 months post-acute onset. The middle column in each panel depicts the mean of 3 tests in the 6–12 months period post-acute onset. The right column represents the mean values of the population of 8 chronic BVL subjects (mean age 44 years), all of whom had velocity values >95% upper limit of 54 normals of mean age 49. This limit, 11 deg/s for pitch and 8.4 deg/s for roll velocity, is shown by the horizontal arrows on the plot ordinates. The bars on the columns represent the sems. ^*^p < 0.03 for the comparison of acute to chronic BVL pitch velocity means. #*p* < 0.003 for the comparison of roll velocity means. Lower panel: sensory analysis of stance profile over the 12 months follow-up of the acute BVL subject. The estimated contribution of each sensory input to pitch sway was computed using the technique of Horlings et al. (9). For example, for the 4 2-legged stance test conditions normal floor eyes open and closed (s2eo, s2ec) and foam support eyes open and closed (s2eof, s2ecf) the visual contribution is estimated to be: ((s2ecf-s2eof) + (s2ec-s2eo))/(s2ecf+s2eof+s2ec+s2eo)^*^100%. The somatosensory contribution is estimated to be: ((s2ecf-s2ec) + (s2eof-s2eo))/(s2ecf+s2eof+s2ec+s2eo)^*^100%. The vestibular contribution is estimated to be 100% minus the visual and somatosensory contributions. The 95% upper limit for visual and somatosensory contribution estimates with respect to healthy controls are shown by the horizontal arrows on the ordinate (larger values pathological), likewise the lower normal 5% limit (smaller values pathological) for the vestibular contribution. Thus, the visual and somatosensory contributions were pathologically greater than normal from 1.3 to 11.7 months and therefore the vestibular contribution less than normal over this period.

The outcome measurements of each balance control trial were the peak-to-peak ranges for roll angle (ra), pitch angle (pa), roll angular velocity (rv), pitch velocity (pv), and the task duration (dur). We concentrated on 2 primary measures, a global balance control index with (BCI) and without stairs test (BCIns) to track improvements in balance control of the acute BVL patient over time. This index combines results from several different tasks into one index (see details below). As secondary measures, we examined trunk sway for those eyes closed tasks which comprise this index. The BCI is an additive composite score based on measures from several tests: From the test standing on 2 legs on foam with eyes closed (2 × pv), for walking 8 tandem steps (1 × ra), for walking 3 m eyes closed (1.5 × pv + 20 × dur), walking 3 m while pitching the head up and down (1.5× pv) and stairs (12× ra). That is BCI = 2 × *s*2*ecf*_*pv*_ + *tan*8_*ra*_ + 1.5 × *w*3*ec*_*pv*_ + 20 × *w*3*ec*_*dur*_ + 1.5 × *w*3*hp*_*pv*_ + 12 × *stairs*_*ra*_ (28). For the first examination, the acute BVL patient could not complete the stairs task so we also used the same index without this task (BCIns). The step-wise discriminant analysis used to select the above task measures entering the BCI is described in Allum and Adkin (8). This combination of the selected balance outcome measures has been shown previously to have a high accuracy in detecting patients with impaired balance (8). The upper 95% limit of the BCI, BCIns, and secondary sway measures of 54 healthy persons of the same mean age (±5 years) as the patient were used to determine if the patient had pathological balance control (see also arrows on ordinates of Figures 2, 3, **5**). We also compared the balance measures of the acute BVL patient with those of 8 chronic (over 10 years) male BVL patients of mean age 44 years whose data had been recorded in previous studies (29, 30). All BVL patients had bilateral absent responses or response < 3 deg/s slow phase eye velocity (SPV) during caloric culmination periods. There was no difference between the rotating chair response amplitudes to 20 deg/s^2^ accelerations of the acute BVL patient (mean SPV 3.9 and −2.6 deg/s) compared to the chronic BVL group (mean SPV 3.2 and −3.2 deg/s).

To compare the difference between the repeated acute BVL and chronic BVL patient population balance measures, and differences in acute BVL vHIT measures over time, both paired *t*-tests and non-parametric paired analyses (Wilcoxon signed rank tests) were used. Significance level was set at *p* < 0.05, and significance was accepted if both tests were significant. We also examined whether individual test values of the acute BVL differed from those of the chronic BVL population using the techniques described by Crawford et al. (31), yielding trends in differences (0.1 < *p* < 0.05) where significance was observed with paired *t*-tests.

## Results

### At Acute Onset

The lateral plane vHIT responses gains on emergency inpatient admission were less than normal, 0.43 and 0.27 for left and right head impulses, respectively. There was also a spontaneous nystagmus beating to the left. These gains did not differ, significantly, from vHIT gains (0.45 left, 0.11 right) recorded for lateral canal planes 4 days later (see Figure 1). Covert and overt catch-up saccades were still present in the vHITs after 4 days. The left beating spontaneous nystagmus was still present having a SPV of 4.4 deg/s. In addition, an upbeat nystagmus with a SPV of 2 deg/s was observed. The initial diagnosis of an acute peripheral BVL could be confirmed by the absent responses to caloric irrigation of 44°C of each ear. After subtraction of the spontaneous nystagmus level, the average SPV over the culmination phase was only 1–2 deg/s (see Figure 1), making computation of the canal paresis of limited use. On admission, a 2-dimensional FLAIR magnetic resonance imaging (MRI) did not show a perfusion deficit or any other abnormality in the brainstem or the cerebellum. A follow-up 2-dimensional CISS MRI, 7 months later, also did not show any signs of a preceding stroke explaining the deficit in this patient. Despite the obvious peripheral loss, functional balance testing revealed results with values indicating better stability than we had expected. The balance control index (BCI), a combined index from several balance tests (8) was slightly larger than normal (see Figure 2) when the stairs test, which the patient could not complete, was excluded. The primary reasons for the lower (more normal) than expected BCI were firstly the almost normal pitch trunk sway velocity when standing eyes closed on a firm and foam surface (see Figure 3). Based on values from the group of chronic BVL subjects we had expected larger trunk sway amplitudes (see Figure 3, upper panels). Secondly, for the walking trials over 3 m, with head rotating left and right, pitching up and down or walking with eyes closed (see **Figure 5**), normal trunk sway amplitudes were observed. Nonetheless, the durations of these gait trials were longer than normal. [Typically for acute UVL patients both trunk velocity and task duration are greater than normal (8) as they attempt to improve stability by walking slowly (32)]. These results indicated together with the greater than normal dependence on visual inputs during stance (see lower panel Figure 3), a more rapid compensation than has been observed with acute UVL patients having an almost total unilateral peripheral loss (5).

**Figure 4:**
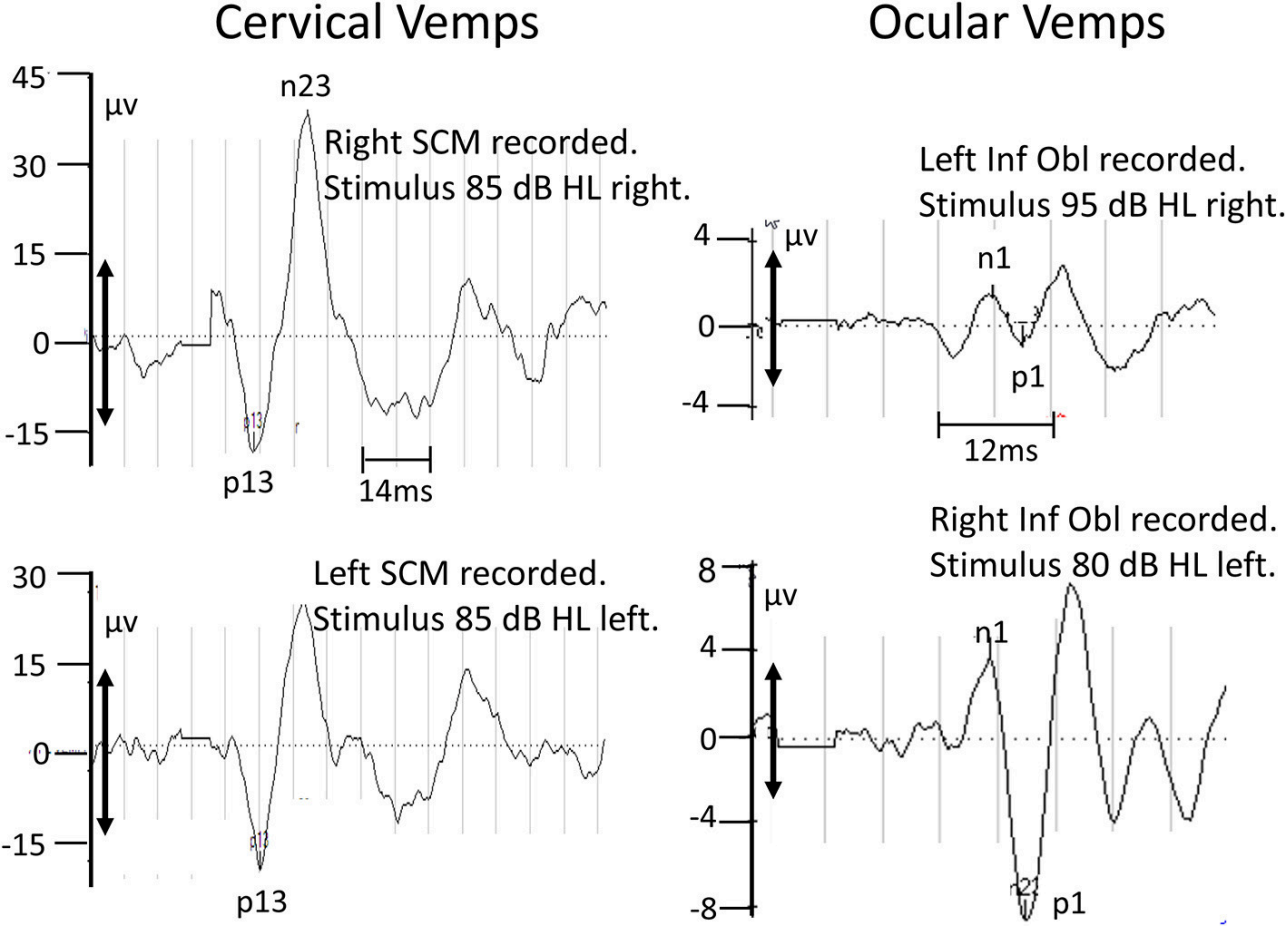
Recordings of c-VEMPs (left panels) and o-VEMPs (right panel) taken 1 and 6.9 months after acute onset of symptoms, respectively. The upper 2 sets of traces are for right ear stimulation, the lower 2 sets for left ear stimulation. The air-conducted (500 Hz) stimulation level was increased to 95 dB to elicit right o-VEMPs. The double-headed arrows on the ordinates indicate the minimum VEMP amplitude (28.6 μV for c-VEMPs, 5.2 μV for o-VEMPs) necessary to define a normal response (above the lower 5% limit of normal control subjects; (27)]. Based on these criteria the right o-VEMPs are not normal.

Tests of c-VEMP indicated that sacculus-driven vestibular spinal reflexes were normal (see Figure 4, left panels). Tests of o-VEMPs carried out at 6 months were normal for the left with lower peak-to-peak amplitudes for the right stimulation side.

### Improvement Over Time

**Figure 5:**
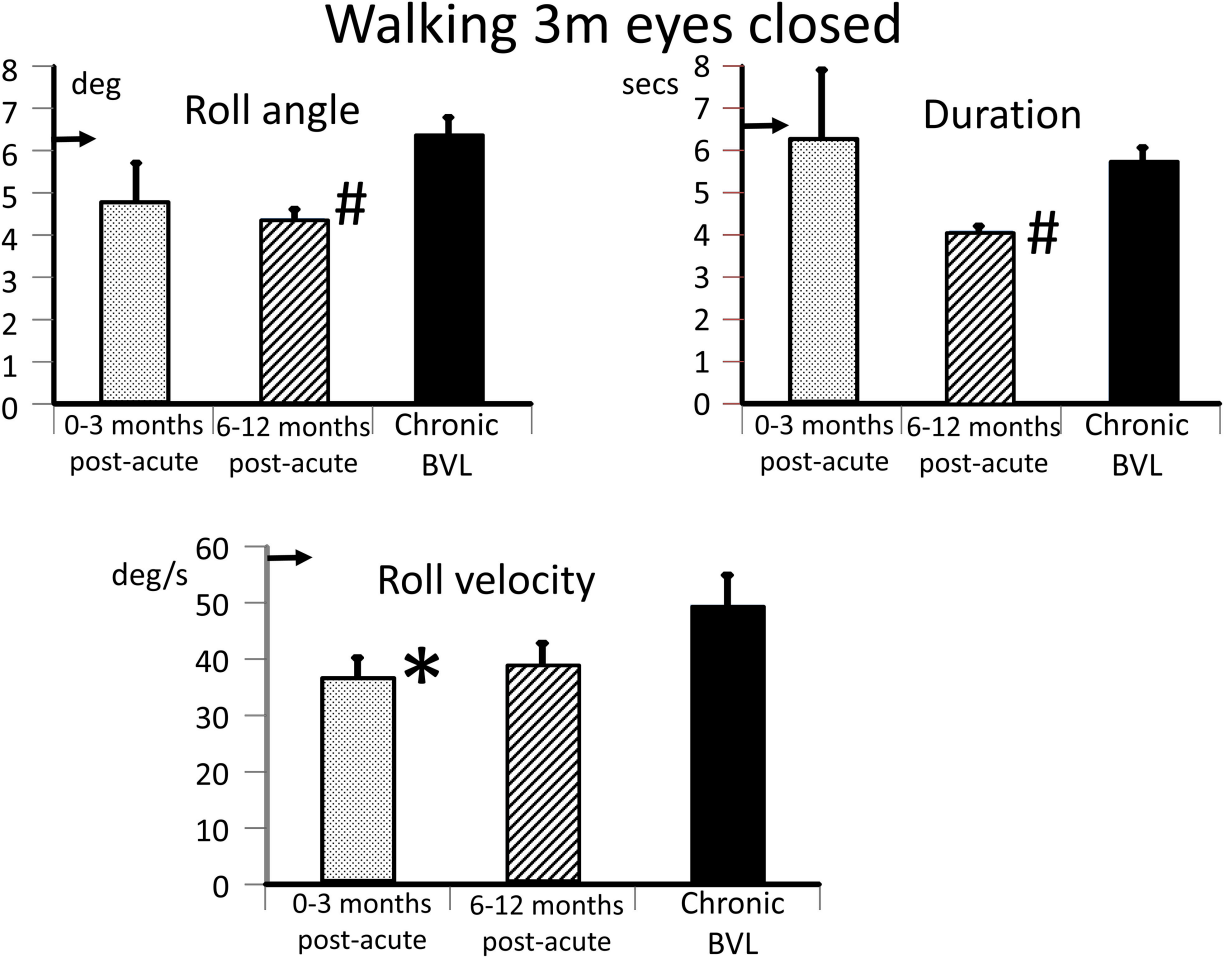
Measures of trunk balance control for the task, walking 3 m eyes closed. The layout of the figure is identical to that of Figure 3 (upper panels). The mean values of the acute BVL subject are compared to a population of 8 chronic BVL subjects and 54 normal control subjects (horizontal arrows on the ordinates). #*p* ≤ 0.002 and ^*^p = 0.05.

The caloric responses did not improve over time but the horizontal spontaneous nystagmus decreased (see Figure 1) and the vertical nystagmus was no longer present. With the exception of the left lateral and right posterior VORs, vHIT response gains increased (see Figure 1) significantly over the 12 months follow-up (*p* = 0.002). However, covert and overt catch-up saccades were still present in the vHITs at 12 months. vHIT gain was below 0.6 bilaterally over the first 9 months [fitting the consensus definition of BVL (6)] and with the exception anterior right (0.63) remained below 0.6 at 12 months (see Figure 1). Balance control as summarized by the BCI value remained on the borderline of normal but was < 95% limit of normal control subjects after 3 months (see Figure 2). This was partially due to sway during stance eyes closed on foam decreasing over time, remaining significantly less (*p* < 0.03) than the sway of chronic BVL subjects (see Figure 3). There was also a gradual decrease in the visual and increase in the somatosensory contribution to stance determined from pitch sway velocities during stance tasks (see Figure 3). However, at 9 months visual and somatosensory contributions were within normal bounds and at 12 months vestibular contributions were normal too (see Figure 3). Another reason for the reduction of the BCI was the increase in gait speed with trunk roll sway angles and velocities remaining normal. For example, as illustrated in Figure 5, task duration for the eyes closed gait task was significantly reduced (*p* < 0.002), with respect to chronic BVL subjects, over the last 6 months of the 12 months follow-up period, as was the roll angle amplitude.

These results match the symptoms reported by the patient and the improvement in balance control reported by his physiotherapist. Oscillopsia reported by the patient was first reduced at examination 5 (at 6.8 months) and absent at examination 6 at 9.4 months. Vertigo and imbalance with fast head movements reported by the patient were also absent at 9.4 months. Persistent problems still noted by the patient were walking in the dark and at dusk. Furthermore, a loss of orientation occurred when swimmingunder water.

## Discussion

This case study raises questions about examination strategies for balance control deficits for patients with suspected vestibular loss. The most important questions are firstly how extensive should testing for sensory deficits be, and secondly at which time points should functional testing of vestibulo-spinal influences on balance control occur. This case emphasizes the viewpoint that VEMP tests of otolith function are crucial for assessing remaining vestibular function in BVL patients (22). We assume that functional balance testing will be required regularly as changes in balance control following a vestibular deficit cannot be predicted, with the possible exception of roll instability during gait (13), by the sensory vestibular loss observed in canal-based responses (7, 11).

This case study indicates that it is crucial in cases of BVL to measure ocular and cervical vestibular evoked myogenic potentials (o- and c-VEMPs) in order to determine if utricular and saccular driven vestibule-spinal reflexes are functioning normally. In our acute BVL patient, VEMPs showed little abnormalities (see Figure 4) despite absent caloric, and lower than normal vHIT VOR canal gains. Remarkably, with apparent preservation of otolith function, the patient was able to stand, without falling, eyes closed on foam for the test time of 20 s within 1 month of acute symptoms, having, except at acute onset, none of the typical stance instability as indicated by higher trunk angular velocities shown on foam surfaces by our group of chronic BVL patients (9). Also, the acute BVL patient was able to walk quicker and more securely after 3 months with eyes closed than chronic BVL patients. These 2 tests together with other stance and gait tests form the basis of functional balance tests identifying patients with vestibular loss (10). As visual compensating inputs are not available during these eyes-closed tests, the most parsimonious explanation for the normal stance and gait performance 3 months after acute BVL onset is that the otolith inputs had been “reprogrammed” to partially replace canal-based vestibular sensory contributions to balance control. It is therefore of interest to consider the basis of the current neurophysiological differences underlying the central adjustments to deficits in semicircular canal responses.

O- and c-VEMPs have been studied before in BVL patients (27, 33). Brantberg and Löfqvist (33) reported preserved c-VEMPs in 5 patients with bilateral vestibular areflexia, in 3 of them there was no significant caloric response, no per-rotatory nystagmus and clinical head-impulse-tests showed corrective saccades following horizontal and vertical head-movements (in two patients head-impulse-tests were not performed). Brain-MRIs of these patients were unremarkable. The recent findings of Agrawal et al. (27) on BVL patients suggest preservation of sacculus and utriculus function relative to semicircular canal function in approximately 40% of BVL patients with decreased function due to aminoglycoside ototoxicity or bilateral Ménière's disease (27). In other BVL cases not described by Agrawal et al. (27), those with vestibular neuritis causing sudden, severe and long-lasting vertigo, the lesion is presumed to be located in the vestibular nerve with a preference for sparing of the inferior nerve due to anatomical considerations (34–36). A bilateral neuritis of the superior vestibular nerve which spared sacculus function (22) can therefore be explained by such anatomical considerations. In our case with bilaterally intact c- and almost intact o-VEMPs alternative explanations must be sought.

An interesting aspect of our observations of this case is the presence of a left beating spontaneous nystagmus (SN), consistent with the slightly greater response to warm caloric irrigation on the left, and the greater lateral canal vHIT gain on the left (see Figure 1). Kattah (37) reported that for subacute and chronic BVL gaze evoked SN is generally not present. We assume that if the degree of loss is unequal between the left and right lateral canal nerves, then a left beating SN would result. Four days after 1st symptoms a weak up beating vertical SN (2.5 deg/s) was also observed. In the following weeks no vertical SN was observed in our recordings. We assume that this vertical SN is probably the result of bilaterally differing vertical canal VOR gains. Initially, the anterior canal vHIT gain was lower on right side compared to left, and the posterior canal gain was lower on the left side compared to the right (see Figure 1), indicating a greater effect of the underlying disease on the right superior nerve and the left inferior nerve. However, the presence o-VEMPs on the right side would tend to exclude a case of pure right superior nerve neuritis. Yacovino et al. (22) reported an upbeating SN with fixation removed in their case of bilateral superior nerve loss suggesting a difference between the peripheral vestibular losses left and right. However, in their case, which they assumed the SN resulted from vestibular neuritis, o-VEMPs were initially absent bilaterally. Likewise, in our case the presence of c-VEMPs bilaterally would tend to exclude a left inferior nerve neuritis. While we assume that differences in peripheral loss bilaterally underlies the left and up beating SN we observed in our BVL case, we cannot exclude that changes in central processing partially underlie the presence of the observed SN. In short, if we assume that our results are consistent with a concomitant vestibular neuritis, we would have to assume that otolith nerves were spared. Confirmatory evidence for this mechanism needs to be acquired from several patients. Here our patho-physiological observations are limited to one patient and do not include 3-dimensional MRI procedures to visualize affected nerves.

Recent advances in 3-dimensional MRI procedures (38, 39) have suggested that it may be possible to visualize which nerves are affected by vestibular neuritis. To date these techniques have been limited to showing that the duration of SN is longer when a higher signal intensity was present on the deficit side (39). In future, use of such MRI signal enhancement techniques in combination with physiological recordings (spontaneous nystagmus, vHIT, VEMPs, and functional balance control examinations as reported here) could provide information on which vestibular nerves were affected following acute BVL or UVL and thereby provide new insights into the etiology of patients' balance deficits and bases for rehabilitative treatment.

The average increase in vHIT gains over 12 months for all 3 test axes for both ears was 0.23 with most of the gain change occurring over the first 7 months (see Figure 1). This value is similar to the increase in lateral vHIT gain, 0.19, observed after 3 months for acute UVL patients who had no caloric recovery from a lateral canal paresis of >90% (5). For the lateral canals, the unchanged CP values indicated that this improvement must be due to central compensation. For the vertical canals, there is no known way to establish whether any peripheral recovery occurred. It is assumed that central compensation with a unilateral loss occurs through a reweighting of the normal contralateral input to the deficit side (3, 4). In the case of bilateral loss, this mode of compensation would appear to be limited. An alternative mode of compensation would be to increase the gain of cervico-ocular reflexes which are known to be increased following BVL (40). Nonetheless, the central compensation of canal VOR responses appears to be modest in our acute BVL patient in comparison to the spared otolith function.

The overall response characteristics we observed are similar to those observed with canal plugging in animals. This preparation inactivates the semicircular canals but not the otoliths. Subsequent testing of the animals indicated that postural stability mainly requires otoliths inputs (14, 15), as we found in our patient.

One theory of central compensation that has been proposed (41) is based on otolith responses being processed by a different portion of the vestibular nuclei (caudally) than canal responses (rostrally). According to this theory, the caudal vestibular nuclei have a greater capacity for sensory substitution. A further possibility is that the caudal vestibular nuclei are more capable of enhancing remaining otolith input than the rostral nuclei are for the modest improvement in canal sensory inputs leading, as illustrated in Figure 1, to only a modest improvement in VOR gains.

One drawback of the current study is that the follow-up period of 1 year may have been too short to determine the long-term result of acute BVL due, as we assume, to vestibular neuritis. Most studies including our own (29, 30) investigated patients with chronic BVL lasting over 10 years. Thus, the differences we observed between the acute and chronic BVL patients may have been due to this short follow-up interval. It is important to note, however, that in this acute BVL case otolith responses were preserved as these were in 40% of BVL patients with other etiologies (27). Another problem with comparing chronic (over 10 years) and recently acute BVL patients is that changes in test procedures occur over time making comparisons difficult, for example, for more recently introduced procedures such as vHIT and VEMPs.

The role of prior training in the use of otolith inputs, due to being the crane cockpit and receiving off-axis rotation may have positively influenced the rapid recovery of the patient. For example, both figure skaters and gymnasts have superior interpretation of otolith signals when no canal signal is present (42, 43). With the preserved otolith responses we observed, the patient could have benefitted from a prior learning to recognize head rotation using otolith signals. This learned strategy may have been reinforced by the intensive physiotherapy the patient received.

Testing for the presence of VEMPs would seem to be crucial in aiding the patient's vestibular rehabilitation in physiotherapy. Further periodic testing of balance control while the patient is receiving physiotherapy provides important information for physiotherapists and physicians needing to base the decision to allow the patient to work again on functional balance control tests. In this report, we have emphasized testing for deficient vestibular contributions to balance control using body-mounted sensors recording trunk sway during functional stance and gait tasks. Other techniques such as dynamic posturography combined with electro-myographic recordings can also be employed (44), but are generally more complex.

## Ethics Statement

Scientific use of the data collected for this study was approved (Approval 2014-16) by the local ethics committee responsible for the University Hospital Basel [Ethics Committee Northwest and Central Switzerland (EKNZ)].

## Author Contributions

JA and FH analysed the data. JA wrote the first draft of the manuscript. FH and HR revised the manuscript. All three authors contributed to the selection and planning of patient tests.

### Conflict of Interest Statement

JA and FH worked as consultants for the company manufacturing the balance measuring equipment used in this study. HR has received travel support from Bayer Healthcare, Teva, and Genzyme.

## Footnotes

^1^Due to the unpleasant patient reactions we do not use ice water calorics. Instead we use rotating chair tests, with accelerations of 20 and 40 deg/s^2^, to determine if there is a remaining peripheral function. In the current case, this test revealed, 4 days after BVL onset, amplitudes of 4.9 and 0.7 deg/s for right and left slow phase eye velocity, respectively, compared to the lower limit of normal responses 20 deg/s (mean-2sds) for20 deg/s^2^ accelerations.
